# Barriers to breastfeeding are shaped by sociocultural context: an exploratory qualitative study in Bangladesh

**DOI:** 10.1186/s41043-022-00312-y

**Published:** 2022-08-13

**Authors:** Md. Fakhar Uddin, Ishrat Jabeen, Mohammad Ashraful Islam, Mahfuzur Rahman, Mohammod Jobayer Chisti, Tahmeed Ahmed, Haribondhu Sarma

**Affiliations:** 1 Nutrition and Clinical Services Division, International Centre for Diarrhoeal Disease Research, Bangladesh (icddr,b), GPO Box 128, Dhaka, 1000 Bangladesh; 2 National Centre for Epidemiology and Population Health, The Australian National University, Canberra, ACT 0200 Australia

**Keywords:** Barriers, Breastfeeding practices, Sociocultural context, Bangladesh

## Abstract

**Background:**

Breastfeeding practice is still not optimum in Bangladesh. Understanding barriers to breastfeeding is needed to prevent harmful practices. This study aimed to understand barriers to breastfeeding among infants and young children in Bangladesh.

**Methods:**

This qualitative study was conducted in five rural sub-districts and one urban slum in Bangladesh. We conceptualized that barriers to breastfeeding can be broadly grouped into individual, society, and system level barriers. We conducted in-depth interviews with 33 breastfeeding mothers and 13 grandmothers of breastfed children (total *n* = 46 interviews). We observed 23 of these infants and young children being breastfed. These data were supplemented by 3 focus group discussions held with the children’s fathers. We managed the data using Atlas.ti software and analyzed the data thematically using an inductive approach.

**Results:**

Important individual-level barriers perceived to influence breastfeeding included misconceptions about the adverse effects of breastfeeding on maternal health, nutrition and physical appearance, and lack of awareness of the value of breastfeeding among family members. Perceived society-level barriers included sociocultural norms, beliefs, and practices such as mother obliged to give more attention on household chores than breastfeeding to become a good housewife and feeding formula milk perceived as a symbol of parents’ financial solvency in the society. System-level barriers included attractive advertisements of breastmilk substitutes, and inadequate facilities and support processes in mothers’ work environments.

**Conclusion:**

A range of barriers at individual, society and system level have important implications for infant and young children’s breastfeeding practices in Bangladesh. Development of interventions that address the range of barriers that many mothers face is essential to support breastfeeding practices. Potential interventions include strengthening information-giving during interaction between mothers and health workers on breastfeeding techniques, and engaging fathers and other “significant others” in counseling on breastfeeding.

**Supplementary Information:**

The online version contains supplementary material available at 10.1186/s41043-022-00312-y.

## Background

The World Health Organization (WHO) has recommended giving breastmilk or colostrum to newborn babies within one hour of birth, feeding exclusively with breastmilk for up to 6 months of age, and continuation of breastfeeding along with appropriate complementary foods up to 2 years [1]. Feeding colostrum to newborns boosts their immune system, exclusive breastfeeding controls gastrointestinal infections, pneumonia, and urinary tract infections, and continued breastfeeding provides appropriate amounts of key nutrients, such as energy, protein, fat, vitamins, and minerals [1–4]. Recommended breastfeeding practices are also cost-effective for families as they are not required to buy breastmilk substitute [5–7]. Given these facts, during the world breastfeeding week of 2018, a target of 50% of mothers achieving optimum breastfeeding practices globally by 2025 was declared to prevent all forms of malnutrition [8].

Despite the positive effects of breastfeeding, current rates of optimum breastfeeding are low around the world. For example, according to the global breastfeeding scorecard, only 23 of 194 countries have exclusive breastfeeding (EBF) rates above 60% [9]. Among South Asian countries, EBF is highest in Sri Lanka (75.8%) and lowest in Pakistan (37.7%) [10]. In Bangladesh, current EBF practices are reported at 55% [11], which is an increase from 28% in the 1990s [12]. However, EBF trends have fluctuated from year to year, at 42% in 2004, 43% in 2007, 64% in 2011, and 55% in 2014 [11]. The median duration of EBF was 2.8 months [11]. In Bangladesh, 17% of children were fed colostrum in the 1990s, increasing to 51% in 2014 [11, 12].

Since 1989, the Bangladesh Breastfeeding Foundation (BBF) has been supporting breastfeeding activities under the primary healthcare system in hospitals and through media [13, 14]. The Government and the BBF contributed to the adjustment of the national code of marketing of breastmilk substitutes in 1993, the introduction of Baby-Friendly Hospital Initiative in 1991, the Maternity Leave Law in 2001, and the policy of optimal duration of EBF for 6 months (180 days) in 2003 [13, 14]. In 2011, the Ministry of Health and Family Welfare (MoHFW) prioritized the infant and young child-feeding (IYCF) intervention and have been implementing the IYCF intervention with non-government organizations-NGOs (e.g., SPRING USAID [Strengthening, Partnerships, Results, and Innovations in Nutrition Globally; United States Agency International Development], Shiree, Surjer Hashi (SH), Concern World Wide, and BRAC—Bangladesh Rural Advancement Committee) under the leadership of the Institute of Public Health Nutrition (IPHN) [15].

Understanding barriers to breastfeeding is needed to prevent harmful practices. In high income settings, marketing of infant formula and short maternity leave policy are barriers [16–18], highlighting the importance of understanding the “market political context.” Understanding the market political context requires investigation and analysis of the entanglement of markets with social and political influences or drivers of behavior. In low income settings, inadequate access to knowledge, delivery outside the health facility, and being psychologically unprepared have been identified as barriers to breastfeeding [19, 20]. In Bangladesh, there are ongoing barriers to breastfeeding despite the implementation of the breastfeeding-promotion programme [21–31]. One study found that the following factors were associated with significantly higher likelihood of initiating breastfeeding within one hour of birth: mothers giving birth in district hospitals, having their visual privacy maintained in the delivery room, newborns crying spontaneously, and newborns being placed in skin-to-skin contact with mothers [32].

The available broader literature suggests that important barriers to breastfeeding can be identified across three inter-related domains: individual-level, society-level, and system-level [22–24, 27, 29, 30, 33]. Previous qualitative studies in Bangladesh identified individual-level barriers as a lack of maternal education, inadequate maternal food consumption, mothers' household workload, time constraints and tiredness/physical weakness of working mothers, inadequate maternal knowledge, and a lack of knowledge among proxy caregivers on feeding stored breastmilk [22–24, 27, 29, 30, 33]. Society-level barriers identified included socioeconomic status of households, gender-based biases, a lack of religious knowledge, generational shifts in feeding behavior, and traditional beliefs [22–24, 27, 29, 30, 33]. System-level barriers included the adverse effects of caesarian delivery, lengthy infant cleaning time at the facility, health workers’ advice to feed formula milk, and maternity leave of less than 6 months [22–24, 27, 29, 30, 33]. Some of the limitations of these qualitative studies were: study participants’ selection, including data collection primarily from young, adolescent or extremely poor breastfeeding women from a few nutrition intervention programme areas—mostly in urban slums [22–24, 27, 29, 30, 33, 34]. As a result, previous study findings and associated intervention designs may not be transferable to or optimal for other settings in Bangladesh. More in-depth exploration of barriers to breastfeeding practices is needed from the perspective of breastfeeding mothers with diverse characteristics (e.g., occupation-employed/housewife; age; education-literacy, geographical location, etc.) and their family members. As part of a larger evaluation of a home fortification with micronutrient power (MNP) programme in Bangladesh [35–37], we conducted this study aimed at understanding the barriers to breastfeeding among a wide range of mothers of infants and young children.

## BRAC’s Maternal, Infant and Young Child Nutrition (MIYCN) programme

BRAC is the largest NGO in Bangladesh, contributing to the improvement of IYCF practices through community health workers (CHWs). The Maternal, Infant and Young Child Nutrition (MIYCN) Phase II was a large community-based home fortification of food with MNP (micronutrient power locally branded as *Pushtikona*) programme implemented by BRAC from 2014 to 2018, targeted to reach about 15 million children aged 6–59 months in 5 years. BRAC implemented the programme nationally in 164 rural sub-districts and in 6 urban slums in Bangladesh. The programme specifically targeted the use of MNP among children aged 6–59 months by a cadre of female volunteer CHWs called *Shasthya Shebika (SS)* and paid CHW called *Shasthya Kormi (SK)*. The roles of CHWs in implementing the MIYCN Programme include selling MNP sachets at the household level and providing counseling education to the caregivers on the use of MNP. In addition, SS provided counseling to the caregivers of children aged 0–23 months about early initiation of breastfeeding and exclusive breastfeeding and demonstrated breastfeeding to promote optimum breastfeeding practice.

## Methods

### Conceptual framework to explore the barriers to optimum breastfeeding practices

**Fig. 1:**
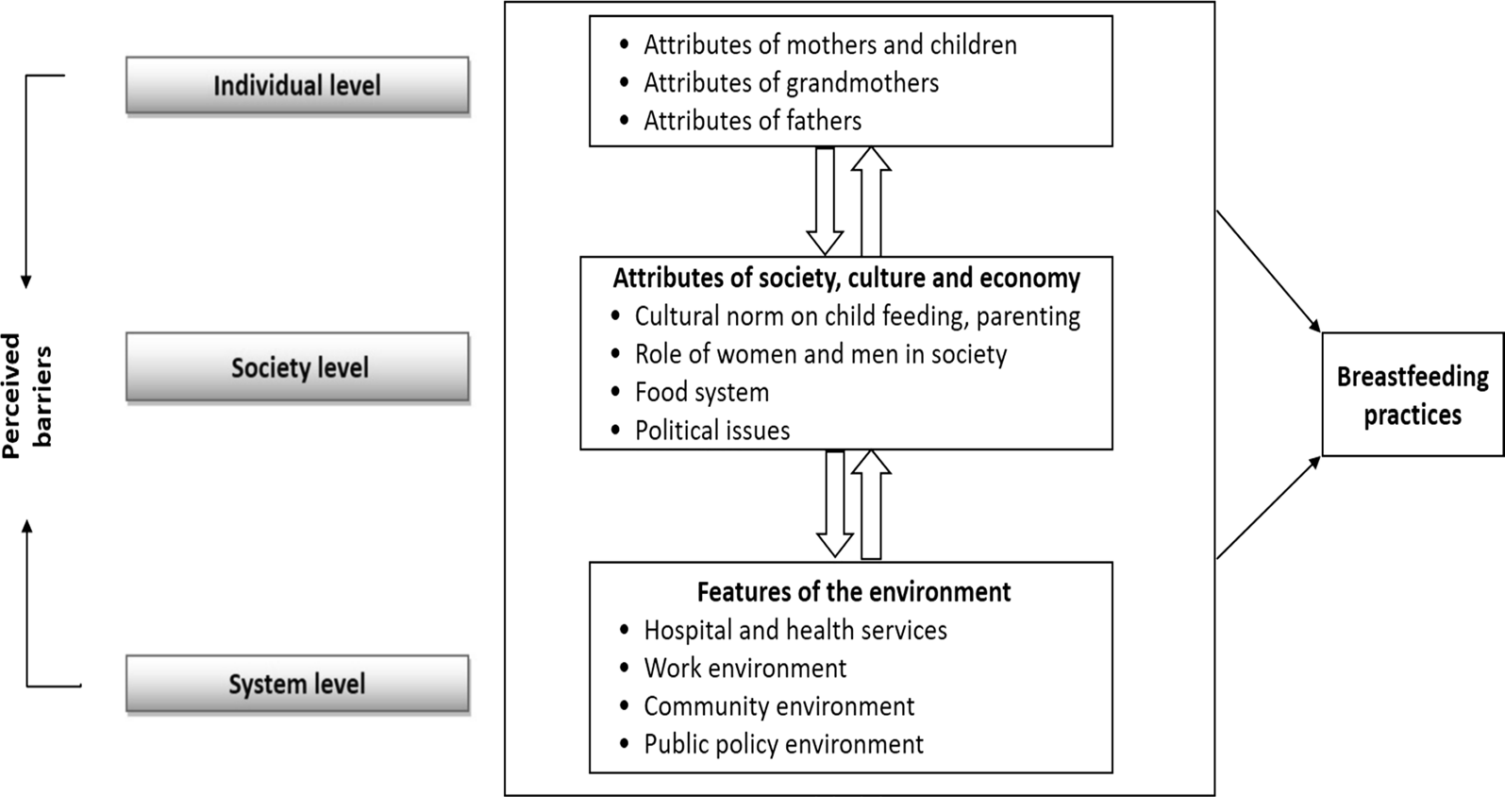
Conceptual framework to explore the barriers of breastfeeding practices

We used a conceptual framework, in Fig. 1, adopted from Hector et al*.* [34] to identify the prevailing barriers to optimum breastfeeding practices. Considering the context of Bangladesh, we conceptualized that barriers can be broadly grouped into three levels: individual, society, and system levels. Barriers at the individual-level are directly related to the attributes of children, mothers, fathers, grandmothers, and other family members. Examples include their intention to practice, behavior, knowledge, skills and parenting experience, as well as perceived health and risk status of mothers and children, and the nature of interactions between the mother and the child. Society-level barriers are related to the attributes of society, culture, and economy. We anticipated that system-level barriers that may influence breastfeeding practices include features of the environment, such as hospital and health services (including resources available, and quality of facilities and of care offered), work environment, and public policy environment.

### Study design

As part of the larger evaluation programme implemented from 2014 to 2018 of the Maternal, Infant and Young Child Nutrition (MIYCN) home fortification programme phase II, we conducted an exploratory qualitative study. A detail evaluation strategy has been published elsewhere [35]. We conducted this qualitative study between June 2015 and May 2016. We collected data through household observations, in-depth interviews (IDIs), and focus group discussions (FGDs) using the semi-structured guidelines.

### Study settings

Data were collected from one urban slum (*Begunbari* within the Dhaka City Corporation) and five rural upazilas (sub-districts) of Bangladesh: Balaganj of Sylhet, Ulipur of Kurigram, Satkania of Chittagong, Monirampur of Jessore, and Gaffargaon of Mymensingh districts. The study sites were purposively selected to include a range of geographical areas covered by the IYCF promotion intervention under the MIYCN programme. We selected more rural than urban study sites because little is known about barriers to breastfeeding practices in rural areas.

### Sampling and recruitment of participants

We purposively selected breastfed children from a range of different age-groups (0–5 months, 6–11 months, 12–23 months) and based on their sex, religion, and mothers’ characteristics (e.g., age, education, occupation). The interviewers went to a community of the selected study sites and then talked to the parents to identify potential children from each age group. We selected eighteen children aged 0–11 months and five children aged 12–23 months, with less children in the latter oldest group as they breastfeed less. The mother, father, and grandmother of the enrolled children were identified as potential interviewees given their likely involvement, experience, and direct or indirect influence on breastfeeding practices. Interviewers approached parents of children without any assistance of the BRAC’s programme personnel. They had no relationship with participants prior to study commencement, and participants were not familiar with the researcher or interviewers. Nobody refused to participate in our study. Initially, we planned to recruit a total of 30 children with an equal number (five) from each study site. However, the final sample size was based on achieving data saturation (followed by criteria of COREQ checklist-additional file 1).

### Data collection

#### Observations

Children of consenting parents were observed in their households to understand breastfeeding practices in real-life settings. A female observer spent a whole day with a mother and her child -she walked with them, observed them, and talked to the mother about her breastfeeding practices. Prior to the observation, mothers were informed that their breastfeeding practices will not be evaluated but that the purpose of this observation was to gain a better understanding of real-life feeding behaviors to support strategic decisions for the health and well-being of other infants and young children in Bangladesh. The observer used a semi-structured observation checklist to capture the observations, which was pilot tested. Detailed notes were taken by the observer in her diary. Mother–child interactions, mothers’ difficulties in breastfeeding her child, breastfeeding time, place, and setting were documented.

#### In-depth interview

Mothers and influential grandmothers of purposively selected children were interviewed to understand their perceptions regarding barriers to breastfeeding. We conducted the IDIs separately to minimize the grandmother’s influence on the mother’s interview. Interviews were conducted at a convenient time for the respondents using a flexible semi-structured guideline which was pilot tested. All interviews were audio-recorded and transcribed verbatim. The average duration of interviews was 65 min.

#### Focus group discussion

A moderator conducted three FGDs with fathers of the selected children (total of 23 fathers) to supplement household observation and IDI data. The average duration of the FGDs was 55 min. The FGDs were conducted at a convenient time and place for the respondents using a flexible semi-structured guideline. All FGDs were audio-recorded and transcribed verbatim.

The data collection team comprised nine experienced icddr,b (International Centre for Diarrhoeal Disease Research, Bangladesh) staff with a background in Anthropology and Sociology. The interviewers were a mix of men and women who spoke the participants’ local dialects. The PI conducted a two-week intensive training for newly recruited team members before data collection. Transcripts were not returned to the participants for their comments or suggestions due to time and budget constraints.

### Data analysis

Recorded interviews and FGDs were transcribed verbatim and detailed observation notes written up. These formed the basis of a discussion with the study’s lead—first author (MFU) and Principal Investigator (PI, last author HS) to obtain immediate feedback to revise the data collection guideline to collect more in-depth information during the next interviews. On completion of the data collection, the transcriptions and observation notes were then carefully read by the data collection team, who coded data in a Microsoft Word file using an inductive approach to develop an initial code list. The initial code list and a deductive code list (developed based on the study objective and data collection guideline) helped to develop a final code list to code all transcribed and written data using Atlas.ti (v.6.2) software. The study lead (MFU) and PI (HS) coded each transcript, compared the results, and resolved any discrepancies. We combined a thematic coding approach and a narrative approach in the analysis of data [36], drawing on our conceptual framework (Fig. 1) and looking for patterns of similarity and difference across rural and urban areas. Here, thematic coding supplemented and enriched the narrative analysis.

### Ethical approval

The Ethical Review Committee of icddr,b approved the study. We had taken informed written consents or thumb impressions (for non-literate participants) before conducting interviews. We assured participants that their information would be used solely for research purposes, and that their name or any other identifying information will not be used in any report or publication. The confidentiality of the information provided by participants was strictly safeguarded.

## Results

In total, 33 children from 33 households were enrolled in the study. Immediately after their enrollment, we observed 23 for a day. We conducted IDIs with 33 mothers and 13 grandmothers, and 3 FGDs with 23 fathers of the enrolled children.

### Background characteristics of children and study participants

The children were mostly Muslim (*n* = 29), with a few Hindus (*n* = 4), and their average age was 14 months. Mothers and grandmothers of children were mostly from rural areas (*n* = 29 mothers; *n* = 10 grandmothers), with average ages of 28 and 59 years, respectively. There were eighteen non-literate mothers, nine mothers who could only sign their name, five mothers who completed primary education, and one mother who completed secondary education. The majority of mothers (*n* = 21) were housewives, with some working as house maids (*n* = 8), Arabic teachers (*n* = 3), and garment workers (*n* = 1). The fathers of the children were mostly farmers (*n* = 12), with some working as day laborers (*n* = 6), rickshaw pullers (*n* = 5), garment workers (*n* = 4), masons (*n* = 2), and electricians, grocery storekeepers, religious leaders (Imam of mosque), and tailors (*n* = 1 each). Table 1 summarizes the demographic characteristics of the enrolled children and other study participants.

**Table 1:** Characteristics of children and their mothers (*n* = 33)

Characteristics	Estimation
Age of children in month (mean)	14
Religion of children (*n*_%)	
Muslims	88
Hindus	12
Residence of children (*n*_%)	
Rural	88
Urban	12
Maternal age (years) (mean)	28
Maternal education (*n*_%)	
Illiterate	55
Could sign only	27
Completed primary	15
Completed secondary	3
Maternal occupation (*n*_%)	
Housewives	64
Maid servants	24
Arabic tutors	9
Garment worker	3

We identified six themes that hinder the breastfeeding practices: misperceptions about breastfeeding; lack of knowledge and skills among family members and mothers; sociocultural norms, beliefs, and practices; cesarean delivery; attractive media advertisements for breastmilk substitutes; and lack of preparedness of workplaces to support breastfeeding. Table 2 illustrates key quotations from study participants to illustrate each theme.

**Table 2:** Key quotations of study participants related to barriers to breastfeeding practices

Broad theme	Sub-themes	Verbatims of study respondents
Individual-level barriers	Misperceptions about breastfeeding	“I did not provide colostrums to my child as I thought, feeding colostrums might cause loose motion as it is very thick, and the newborn infants are unable to digest it. Therefore, I gave honey to my child.” [140429M01_IDI_MO_Stk_Ctg_rural] “My grandson was born at a delivery centre and immediately after the birth, I provided sugar-mixed water to the newborn child concealing the view of the nurse. I thought that the child was crying because of his appetite when the mother was unable to produce breastmilk.” [140530M01_IDI_GM_Bgb_Dha_urban] “I provided honey to my grandson immediately after the birth as I thought that feeding honey first will facilitate to behave well at his adult age.” [140429M01_IDI_GM_Stk_Ctg_rural] “I thought that water should be provided to the child immediately after birth as the child becomes thirsty as he/she comes from one earth to another earth.” [140509M01_FGD_FAT_Gfg_rural] “I have forbidden my wife to provide breastmilk more often as I thought that excessive breastfeeding might be harmful to teeth of the child (can be affected by insect).” [140429M01_FGD_FAT_Stk_Ctg_rural] “I became pregnant recently, and I thought that at this moment if I continue breastfeeding, my baby in the womb will get less nutrition.” [040429M01_IDI_MOT_Stk_Ctg_rural]
	Lack of knowledge and skills among family members and mothers	“I was supposed to provide sugar-mixed water to my newborn baby as per the instructions from my mother-in-law, but I did not know the benefits of giving this.” [140506M01_IDI_MOT_Gfg_rural] “A mother breastfed her child immediately after feeding complementary food when she lies down on the bed and talking to the father of the child. In this condition, the child discontinued taking breastmilk after feeding 2–3 min, then the child started crying and the mother gave chocolate to the child to stop crying.” [140326M01_OBS_Blg_Syl_rural]
Society-level barriers	Sociocultural norms, beliefs, and practices hinder breastfeeding	“My mother-in-law instructed me to provide formula milk to my child when I became busy for cooking meals for other household members. My mother-in-law thought if I breastfeed my child, he/she could take much time, resulting in disruption of cooking meals in time. Therefore, to satisfy her I provided formula milk to my child.” [140429M01_IDI_MOT_Stk_Ctg_rural] “The mother of my grandson often provides semolina instead of breastfeeding because of her lack time. She is mainly responsible for doing household chores as she is the young energetic woman in the family.” [140429M01_IDI_GM_Stk_Ctg_rural] “I gave instruction to the mother of my grandson to limit breastfeeding more often as I knew from elders that excessive breastfeeding is responsible for becoming fool at his adult age.”[ 140429M01_IDI_GM_Stk_Ctg_rural] “I purchased formula milk for my child as most children in our community feed formula milk. It might be beneficial for the health of the child.” [140509M01_FGD_FAT_Gfg_rural] “I provided my child (age 4 months) sweet food received from the mosque as I considered it a blessed food, which might be beneficial to the health of the child.” [140429M01_FGD_FAT_Stk_Ctg_rural]
System-level barriers	Adverse effects of cesarean delivery on breastfeeding	“I had been suffering for the last few days from an infection in my surgical incision mark immediately after my cesarean delivery in a low-cost facility. At that time, I had no appetite for food due to taking a high-power antibiotic for a long time. So, I was unable to produce breastmilk sufficiently, and I initiated formula milk as a substitute for breastmilk. However, after the improvement of my illness, I was still unable to breastfeed sufficiently. In the meantime, the child was habituated to formula milk.” [140530M01_IDI_MOT_Blg_Dhk_rural] “My child was crying much when I became senseless immediately after my cesarean delivery at the hospital. Then, my sister fed formula milk to my child to stop the child’s crying as per the physician’s instruction of the hospital.” [140506M01_IDI_MOT_Gaf_Mym_rural]
	Attractive media advertisements to initiate breastmilk substitutes	“Since I thought that I was unable to produce sufficient breastmilk to feed my child, I went to a physician and requested him to write the name of formula milk. I learned about feeding formula milk from watching television, but I was unaware which one is best for child health. Then, the physician prescribed a name of formula milk; thus, I started feeding formula milk at 4 months of child age.” [140507M01_IDI_MOT_Tithi_Gfg_rural] “I went to a physician when my child was 4 months to receive treatment for the problem of production of insufficient breastmilk. Instead of giving treatment to improve the production of breastmilk, the physician suggested and prescribed a name of formula milk as an alternative of breastmilk.” [140530M01_MOT_Bgb_Dhk_urban]
	Inadequate facilities and support process in mothers’ work environment	“I live in a nuclear family where no one is available to look after my child. In this situation, I am not allowed to work as a maid-servant in others’ households if I want to carry and keep my child with me. Meanwhile, I have lost a few job opportunities because of the situation, but I desperately needed a job to survive.” [140530M01_IDI_MOTi_Bgb_Dhk_urban] “Most women in my community are garment workers. A mother cannot bring and keep her child in the garment factory as the factory owner does not allow as he was concerned about the loss of work time due to breastfeeding of any child, which will reduce the overall productivity of the factory.” [140530M01_FGD_FAT_Bgb_Dhk_urban]

The findings against each theme are outlined in turn below.

### Individual-level barriers

#### Misperceptions about breastfeeding

Despite hearing (from media and CHWs) about the health benefits associated with breastfeeding, many mothers and grandmothers had misperceptions about feeding colostrum to newborn infants. Many mothers believed that it might cause loose stool as it is very thick and newborn infants are unable to digest it. As a result, these mothers gave honey or sugar-mixed water to their newborn babies instead of colostrum, in some cases concealing the practice from health workers. Misperceptions among the mothers also hindered exclusive and continued breastfeeding. A rural undernourished (self-defined) mother of an 8-month-old child said,

“I stopped breastfeeding my child as I thought I will become more bony and physically weak if I continue to breastfeed my child; because the remaining vitamins of my body will be transferred to the child’s body.” [140506M01_IDI_MOT_Gfg_Dha_rural]

Many mothers did not breastfeed their newborn babies when ill as they perceived this might cause illness in the child. Mothers who became pregnant within a short birth interval believed that their baby in the womb will get less nutrition if she continues to breastfeed, which discouraged them to continue breastfeeding. Four husbands (children’s fathers) believed that continued breastfeeding would spoil their wife’s physical shape and ruin their sexual attractiveness. This discouraged their wives from continuing breastfeeding.

#### Lack of knowledge and skills among family members and mothers

Mother reported that they did not follow the breastfeeding advice of community health workers (CHWs) because they felt under pressure to follow the instructions of elderly family members (e.g., grandmother) to give formula milk or semolina to their children aged less than six months. These mothers had expressed their desire to ensure exclusive breastfeeding. The elderly family members forced mothers to feed water to their newborn babies. This happened if the colostrums did not come out from the mother’s breast within a few hours of the birth or if the mother failed to feed the baby. The elderly family members thought that the newborn was thirsty or hungry. Mother stated that they have a lack of understanding of when to breastfeed and breastfeeding position. For example, one mother realized that her child refused to breastfeed after she offered breastfeeding immediately following the completion of complementary feeding. Three mothers recognized that most mothers positioned their children inappropriately while breastfeeding them and watching television by mothers and gossiping with others. Consequently, their children were reluctant to suck the breast and often refused to breastfeed. These mothers blamed insufficient breastmilk production as the children often cried soon after the refusal. In such cases, we observed that the mothers provided their children formula milk instead of breastfeeding. A rural Bangladeshi mother said,

“I visited a physician when my child did not get sufficient breastmilk, and asked him to prescribe formula milk, but he was opposed to giving any formula milk before six months old of the child. However, I insisted on him to write brand name of any formula milk, and he eventually prescribed a formula milk.” [140429M01_IDI_MOT_Stk_Ctg_rural]

Participants (mother, grandmothers, and fathers) reported that first time and adolescent-age mothers were unaware of proper breastfeeding techniques, resulting in insufficient breastfeeding to their children. This occurred in the case of mothers living with their husbands and having a newborn child in a nuclear family in urban slums. These mothers had a lack of interaction with breastfeeding-experienced neighbor mothers. Their children often cried, and the mothers assumed that this was due to getting insufficient breastmilk, which influenced them to give their children formula milk. An urban mother of four-month-old child in Bangladesh said,

“I am not familiar with the experienced breastfeeding mother to learn about the techniques of breastfeeding as I recently moved to this urban slum from a rural area with my husband and child.” [140530M01_IDI_MOT_Bgb_Dhk_urabn]

### Society-level barriers

#### Sociocultural norms, beliefs, and practices hinder breastfeeding

Mothers were considered a good housewife and admired by other family members if they completed other household chores in time. These expectations put pressure on mothers to dedicate more time to household chores rather than adequately breastfeeding their children. As a result, mothers were influenced to feed their children formula milk instead of breastfeeding in order to avoid the inconvenience of breastfeeding and to have more time for doing household chores smoothly. Furthermore, feeding formula milk to children was perceived as a symbol of parents’ financial solvency in the community.

Mothers believed that the traditional practices of serving food to the household’s members hindered adequate breastfeeding practices because they prevented mothers from eating adequate amounts of food in time for adequate breastmilk production. We observed that women in the household, including the lactating mother, had eaten food at the end if food was available after providing food to the male members and kids. Three mothers reported that they were unable to intake enough food due to a financial problem created by a sudden loss of income due to seasonal changes. As a result of insufficient food intake, mothers were occasionally left hungry, which was the perceived cause of poor breastmilk production.

Continuation of breastfeeding was determined by the mother taking into consideration whether her child is male or female. The mothers felt discomfort about continuing to breastfeed their young male children (15–24 months). A mother of 15-month-old male child stated,

“My boy is getting naughtier day by day; he often bites my nipples during breastfeeding, causing me a lot of pain. So, I am planning to discontinue breastfeeding soon. But this was rarely happened to my older female child.” [140530M01_IDI_MOT_Dipti_Bgb_Dhk-urban]

Lack of religious knowledge also influenced breastfeeding practices. Some Muslim mothers of 16 months old children believed that it would be a sin for them if their boy child continues breastfeeding until aged two years old, so they had stopped breastfeeding their children. However, eight religious minded fathers stated that these mothers had lack of knowledge about religious (Muslims) instructions (written in the Holy Quran—continue breastfeeding until two years). Religiously, it does not matter whether the child is a boy or girl.

### System-level barriers

#### Adverse effects of cesarean delivery on breastfeeding

The mothers who gave birth through cesarean section made the decision to start formula milk as soon as the deliveries because they claimed to produce insufficient amounts of breastmilk. During antenatal care (ANC), these mothers were encouraged by a physician to have a caesarian section in a private hospital or in a clinic in order to have a safe delivery and relief from the pain of normal vaginal delivery. Five fathers perceived that these physicians were received incentives from private hospitals and clinics. Two mothers reported that they were unable to produce enough breastmilk as a result of taking high-power antibiotics during the post-delivery period. Some cesarean section mothers exclusively fed their children formula milk instead of breastfeeding their children. One mother said:

“My elder child was born at home [through normal vaginal delivery] and I was able to produce enough breastmilk to ensure exclusive breastfeeding practices. Furthermore, I gave birth to my younger child at a private clinic through caesarian section as per physician suggestion due to my complications during pregnancy. Then, I could not produce enough breastmilk and was obliged to start feeding formula milk before the child aged 6 months. I think that intake high power antibiotic to recovery my condition after cesarean delivery might be the cause of inability to produce breastmilk.” [140533M01_IDI_MOT_ST_Syl_-rural]

#### Attractive media advertisement to initiate breastmilk substitutes

Four mothers reported that they were inspired to choose formula milk by attractive media advertisements on televisions that explained the benefits of feeding formula milk and depicted the picture of a healthy child and a smart mother. Two mothers also reported that they had used contraceptive products by the influence of appealing media advertisements on television and in newspapers which may have reduced their breastmilk production. Furthermore, three mothers were feeding their children formula milk because physicians and pharmacy shopkeepers willingly prescribed and advised the formula milk when mothers visited them for seeking treatment since they were incentivized by the manufacturer company of formula milk.

#### Inadequate facilities and support process in mothers’ work environment

In urban areas, fathers and employed mothers stated that working mothers were obliged to join the workplace (particularly in garment factories) before their children were six months old, despite the lack of a daycare center in the workplace to ensure EBF. They perceived—the establishment of a daycare center at the garment factories as a cost burden and shortage of space which ultimately had discouraged many factory owners to do so. In addition, one father reported that the factory owners perceived—if an employed mother goes to a daycare center to breastfeed her child, it may reduce productivity of the factory. As a result, working mothers were unable to perform exclusive and continue breastfeeding practices.

Few mothers who work at garment factories in urban Dhaka keep their children in their rural houses under the care of their paternal or maternal grandmothers. This strategy enabled working mothers to perform a high volume of work for a good earning without any hassle of childcare. Moreover, two mothers said that they had lost their job due to spending time breastfeeding their babies in the workplace. One mother stated that it reduces the chances of getting a new job. An urban mother of eight-month-old child stated,

“I live in a nuclear family where no one is available to look after my child. In this situation, I am not permitted to work in others’ households if I want to carry my child with me. Meanwhile, I have lost a few job opportunities because of the situation, but a job is very much needed for me to survive.” [140530M01_IDI_MOT_Bgb_Dhk_urban]

Furthermore, two working mothers perceived that feeding their children stored breastmilk is an unusual practice, time-consuming, painful, and hassles to them and proxy caregivers. As a result, proxy caregivers fed formula milk or semolina and often provided low-quality junk food when the mothers went outside for work.

## Discussion

This study identified key perceived individual-level barriers to breastfeeding practices including misperceptions about the adverse effects of breastfeeding on maternal health, nutrition, and physical appearance, a lack awareness of the value of breastfeeding among family members and mothers. Identified society-level barriers include sociocultural norms, beliefs, and practices. System-level barriers include attractive advertisements and advice of health workers of breastmilk substitutes; adverse effects of cesarean delivery on breastmilk production; and inadequate facilities and support process in mothers’ work environment. The results of analysis demonstrated that some barriers that were identified in the last two decades are still remaining and are deeply shaped within the existing sociocultural and market political context. We discuss five important cross-cutting barriers to breastfeeding across three levels: misperceptions about the adverse effects of breastfeeding; lack of awareness about the value of breastfeeding among family members and mothers; sociocultural norms, beliefs, and practices; adverse effects of advertisement and cesarean delivery; and inadequate facilities and support process in mothers’ work environment.

We revealed that many mothers were aware of the benefits of breastfeeding, but some misperceptions about adverse effects of breastfeeding remained that were deeply shaped in other family members; for example, the baby in the womb will get less nutrition if pregnant women continue to breastfeed, and continued breastfeeding would spoil mother’s physical shape and ruin their sexual attractiveness. This could be due to a lack of interaction between family members and health workers or CHW did not provide additional information to correct these misperceptions. Previous qualitative studies in Bangladesh did not explore these misperceptions [27, 29]. However, in line with our study findings, previous studies have revealed a few misperceptions about administering honey, sugar, or water and mustard oil which disrupted breastfeeding practices [27, 29]. We heard informally from a few mothers that they tried to reduce some misperceptions of other family members, but that they often failed as gendered issues persist in children’s households, as detailed elsewhere [37]. As a result, breastfeeding practices are still suboptimal [29, 38, 39]. In this situation, interventions aimed at increasing mothers’ knowledge to promote breastfeeding practices may be ineffective, because other family members’ misperceptions influenced mothers to initiate breastmilk substitute. We believe that potential interventions are needed to promote breastfeeding practices through education of the mother, key family members, traditional birth attendant, and the peer supporters [21]. In this regard, couple counseling or courtyard meeting with family members counseling by CHWs can be potential interventions to educate other family members and reduce their misperceptions.

Many mothers were unaware of the ideal time, location, and environment for breastfeeding. Children refused to feed breastmilk due to the mother’s lack of eye contact with the child, and the noise of gossiping and watching television, whereas mothers assumed this occurred due to a lack of breastmilk production and supply. Previous qualitative research found that low breastmilk production was caused by the mother’s poor health and nutrition, particularly during floods [40]; lack of maternal motivation, confidence, and infrequent suckling [29, 41, 42]; and a shorter duration of breastfeeding [42]. In line with earlier studies conducted in Bangladesh [29, 40, 42–45], we discovered a lack of awareness about breastmilk production among mothers and family members, which led to initiate breastmilk substitutes. The CHW can conduct a weekly/fortnightly meeting with a mother’s group in a convenient location in the community or form a peer supporter women group to train them, particularly the adolescent and new mothers in the community, to ensure recommended breastfeeding practices through practical demonstration and providing necessary information related to breastfeeding time and ideal location. It is worth noting that a previous study from urban Bangladesh discovered that peer counseling from local female volunteer improved breastfeeding practices [46]. However, our study findings strongly suggest that establishing community-based peer support women groups not only in urban, but also in rural areas for counseling to mothers to improve their breastfeeding practices. Previous systematic review [47] showed that it is similarly beneficial in rural areas. When providing breastfeeding advice to mothers, CHW can emphasize the importance of an appropriate location and environment for adequate breastfeeding practices.

Mothers’ household workload—having to juggle multiple domestic responsibilities in addition to breastfeeding for the child, difficulties in getting support from the child’s father and others were influenced to breastfeeding practices and to give breastmilk substitutes to their children. These barriers were often in the context of physical environments and in homes without easy access back to breastfeeding. Exclusive and continued breastfeeding was not possible to ensure by mothers who had pressure from family members to perform household chores on time, eager to become a good housewife, and demonstrate mother’s smartness and financial solvency of the family in the community through buying and giving formula milk. These sociocultural norms that we discovered as barriers to breastfeeding practices had not previously [29, 33] been thoroughly investigated. One study [38] stated that caregivers could not properly feed complementary food to their children due to seasonal household chores, which is also consistent with our study findings regarding breastfeeding practices. Furthermore, we revealed that sociocultural norms influenced inadequate maternal food intake, resulting in insufficient breastmilk production—hampered breastfeeding practices, despite the fact that there were no seasonal effects and the necessary foods were available in their houses. Therefore, we think that CHWs can conduct a family meeting or couple counseling to provide counseling to both mothers and other influential family members in order to improve breastfeeding practices. In addition, we revealed that mothers were unable to eat healthy and appropriate food for adequate breastmilk production due to households’ inability to buy food, which was previously little explored elsewhere [29, 33]. Micro-saving from the first day of pregnancy can ensure their financial security and support during the lactation period to manage the essential healthy daily food items for breastfeeding mothers. Conditional cash transfer may also ensure mothers’ sufficient food intake to produce enough breastmilk.

Attractive media advertisement for feeding formula milk was identified as an important perceived system-level barrier to breastfeeding practices. Previous studies had never looked into this. In Bangladesh, there is a legislation to limit the marketing of breastmilk substitutes [21]. Therefore, it is necessary to strengthen the monitoring of the application of the existing legislation. Furthermore, we discovered that the advice of physicians and local drug sellers had shifted mothers’ behavior and practices from breastfeeding to formula milk feeding their children. Only one study from rural Bangladesh found that village doctors or drug sellers had a greater influence on formula feeding decisions [48], but it was unable to investigate the underlying reasons. According to our findings, low-profit margins from selling formula milk products as well as manufacturers’ withdrawal of incentives for physicians to prescribe formula milk products can reduce formula milk usage and improve breastfeeding practices.

In Bangladesh, infants delivered by cesarean section had lower odds of exclusive breastfeeding than those who delivered normally [28]. Studies have found that psychological distress of mothers, too much pain after giving birth, mothers unconsciousness, lengthy infant cleaning time at the facility, and mothers too weak to breastfeed were the barriers of early initiation of breastfeeding and exclusive breastfeeding practices among the caesarian delivery mothers [27, 30, 33]. Usages of high-power antibiotics and infrequent breastfeeding after having the caesarian delivery led to insufficient breastmilk production and influenced other family members and physicians to advise on feeding formula milk [27, 29, 33, 48], which is also consistent with our study findings. Additionally, we revealed that the mothers who had cesarean deliveries at low-cost private healthcare facilities had many of these challenges. In the context of Bangladesh, some physicians promote cesarean delivery even if it is not clinically indicated as they are financially rewarded by private hospitals or clinics. Cesarean section delivery, particularly in low-quality healthcare facilities, and complications that arise after delivery have become more common over time as a result of the influence of dishonest physicians and mothers from financially secure families. We believe that cesarean section delivery should not be considered in the context of high privatization and low-quality medical services in order to promote breastfeeding practices. A social behavioral-change communication intervention to the pregnant mothers using mass media, counseling, and motivation by CHWs, physicians, and nurses during ANC and delivery periods may also reduce the number of unexpected or non-clinical cesarean deliveries which may have a positive impact on breastmilk production and breastfeeding practices.

Caregivers knew about the benefits of breastmilk, but many stopped it entirely due to maternal employment [29, 30, 44], lack of maternity leave [27], tiredness/weakness of working mothers, and time constraint [30]. More specifically, mother’s shyness to breastfeed in front of others at work place, mothers’ unwillingness to extract breastmilk when they leave for work early in the morning, support caregiver’s (inadequate knowledge) inability to feed the extracted and stored breastmilk in a timely manner were identified barriers of exclusive and continued breastfeeding practices [30, 33, 40]. In addition, we discovered that job providers did not allow mothers to bring their children to the workplace for breastfeeding due to a lack of daycare facilities and a supportive work environment. This influenced mothers to keep their breastfeeding child at home or to send their child to their rural household to work effectively at their workplace. Therefore, the poor working mothers gave a breastmilk substitute to their children as they considered it as an investment to perform better work for a good earning and secure job. Establishing a daycare center in the workplace, revision of leave policy to ensure exclusive breastfeeding, and the flexibility in the working hours in private services especially in garment factories may improve the breastfeeding practices. Counseling and demonstration about extracting breastmilk, storing, and feeding processes are needed to provide to the working mothers and support caregivers.

### Strengths and limitations of the study

There were a number of strengths of this study. We used a number of qualitative data collection methods, including interviews, observations, and FGDs. This helped us to explore depth of information from perspectives of diverse participants and undertake triangulation of findings from different angles. Despite these strengths, this study has some limitations. First, data collection in urban slums was limited to Dhaka city, which may not represent the scenarios of other city slums of Bangladesh. Furthermore, children living outside the slum were not included in this study due to resource and time constraints. As a result, findings of this study only reflect to children of urban slums and rural areas. Second, we only interviewed mothers and their influential family members; however, interviews with the CHWs, physicians, nurses, community elites, and village doctors, or pharmacy shopkeepers could provide further insights. It was not possible due to a lack of time and resource constraints.

## Conclusions

A range of barriers at individual, societal, and system level have important implications for infants and young children’s breastfeeding practices in Bangladesh. Development of interventions that address the range of barriers that many mothers face is essential to support breastfeeding practices. Immediate potential interventions for positive change in breastfeeding practices include strengthening information giving during interaction between mothers or family members and health workers on what breastfeeding techniques are needed to follow by mother—in which settings and why, importance of adequate nutritious food intake of mothers; engaging fathers and other “significant others” in counseling on breastfeeding; strengthening monitoring of the marketing of breastmilk substitute products; considerations of low-profit margins from selling formula milk products and the removal of manufacturer incentives for physicians; strengthening breastfeeding policies for working mothers; and recognizing mothers who follow the recommended breastfeeding practices for their children. Potential intervention strategies could include: weekly or biweekly community meetings with mothers' and influential family members’ groups; family meetings or couple counseling at children's homes; formation of peer supporter women group for breastfeeding counseling and demonstration; micro-saving from the first day of pregnancy or conditional cash transfer to ensure adequate food intake of lactating mothers; and stakeholders consultation meetings. Given intersectional barriers, interventions might be integrated. The structural drivers of women’s position in society and gender inequity must also be addressed in order to maximize and sustain the impact of immediate actions and interventions. This requires interventions to ensure equal equitable opportunities for men and women in all aspects of life, including access to food. Given patriarchal norms locally and globally, men will likely need special targeting and support in achieving optimal breastfeeding practices.

## Supplementary Information

**Additional file 1.** COREQ checklist.

## Data Availability

The date sets cannot be shared publicly due to institutional roles and regulations. Data generated during the study will be provided to interested researchers (recipients) from the corresponding author on reasonable request.

## Abbreviations

ANC: Antenatal care
BBF: Bangladesh Breastfeeding Foundation
BRAC: Bangladesh Rural Advancement Committee
CHWs: Community Health Workers
CIFF: The Children’s Investment Fund Foundation
EBF: Exclusive breastfeeding
FGDs: Focus group discussions
GAIN: The Global Alliance for Improved Nutrition
IYCF: Infant and young child-feeding
IPHN: Institute of Public Health Nutrition
IDIs: In-depth interviews
icddr,b: International Centre for Diarrhoeal Disease Research, Bangladesh
MoHFW: Ministry of Health and Family Welfare
MIYCN: Maternal, Infant and Young Child Nutrition
NGOs: Non-government organizations
PI: Principal investigator
SPRING USAID: Strengthening, Partnerships, Results, and Innovations in Nutrition Globally; United States Agency International Development
SH: Surjer Hashi
UK: United Kingdom
WHO: The World Health Organization
